# Hypermethylation of the alternative *AWT1* promoter in hematological malignancies is a highly specific marker for acute myeloid leukemias despite high expression levels

**DOI:** 10.1186/1756-8722-7-4

**Published:** 2014-01-09

**Authors:** Amy Guillaumet-Adkins, Julia Richter, Maria D Odero, Juan Sandoval, Xabi Agirre, Albert Catala, Manel Esteller, Felipe Prósper, María José Calasanz, Ismael Buño, Mi Kwon, Franck Court, Reiner Siebert, David Monk

**Affiliations:** 1 Imprinting and Cancer group, Cancer Epigenetic and Biology Program, Institut d’Investigació Biomedica de Bellvitge, Hospital Duran i Reynals, Av. Gran Via de L’Hospotalet 199-203, 08907 L’Hospitalet de Llobregat, Barcelona, Spain; 2 Institute of Human Genetics, Christian-Albrechts University, Kiel, Germany; 3 Department of Genetics, University of Navarra, Pamplona, Spain; 4 Cancer Epigenetics group, Cancer Epigenetic and Biology Program, Institut d’Investigació Biomedica de Bellvitge, Hospital Duran i Reynals, Barcelona, Spain; 5 Division of Cancer and Area of Cell Therapy and Hematology Service, Universidad de Navarra, Pamplona, Spain; 6 Servicio de Heamatología, Hospital Sant Joan de Déu, Barcelona, Spain; 7 Department of Physiological Sciences II, School of Medicine, University of Barcelona, Barcelona, Catalonia, Spain; 8 Institucio Catalana de Recerca i Estudis Avançats (ICREA), Barcelona, Catalonia, Spain; 9 Department of Hematology, Hospital General Universitario Gregorio Maranon, Madrid, Spain

**Keywords:** WT1, Methylation, Epigenetics, Leukemia, Imprinting

## Abstract

**Background:**

Wilms tumor 1 (WT1) is over-expressed in numerous cancers with respect to normal cells, and has either a tumor suppressor or oncogenic role depending on cellular context. This gene is associated with numerous alternatively spliced transcripts, which initiate from two different unique first exons within the *WT1* and the alternative (A)*WT1* promoter intervals. Within the hematological system, *WT1* expression is restricted to CD34+/CD38- cells and is undetectable after differentiation. Detectable expression of this gene is an excellent marker for minimal residual disease in acute myeloid leukemia (AML), but the underlying epigenetic alterations are unknown.

**Methods:**

To determine the changes in the underlying epigenetic landscape responsible for this expression, we characterized expression, DNA methylation and histone modification profiles in 28 hematological cancer cell lines and confirmed the methylation signature in 356 cytogenetically well-characterized primary hematological malignancies.

**Results:**

Despite high expression of *WT1* and *AWT1* transcripts in AML-derived cell lines, we observe robust hypermethylation of the *AWT1* promoter and an epigenetic switch from a permissive to repressive chromatin structure between normal cells and AML cell lines. Subsequent methylation analysis in our primary leukemia and lymphoma cohort revealed that the epigenetic signature identified in cell lines is specific to myeloid-lineage malignancies, irrespective of underlying mutational status or translocation. In addition to being a highly specific marker for AML diagnosis (positive predictive value 100%; sensitivity 86.1%; negative predictive value 89.4%), we show that *AWT1* hypermethylation also discriminates patients that relapse from those achieving complete remission after hematopoietic stem cell transplantation, with similar efficiency to *WT1* expression profiling.

**Conclusions:**

We describe a methylation signature of the *AWT1* promoter CpG island that is a promising marker for classifying myeloid-derived leukemias. In addition *AWT1* hypermethylation is ideally suited to monitor the recurrence of disease during remission in patients undergoing allogeneic stem cell transfer.

## Background

Overexpression of the Wilms’ tumor 1 gene (WT1) is implicated in the prognosis of leukemia with high expression predicting disease progression in acute myeloid leukemia (AML), as well as being intensively studied as a potential molecular marker for minimal residual disease (MRD) and treatment response. In the normal scenario, WT1 expression is restricted to CD34+ cells in the bone marrow and absent in peripheral blood cells; therefore expression of this zinc-finger transcription factor is limited to early progenitors of the blood system, suggesting that this gene plays a critical role in controlling proliferation and/or differentiation in hematopoietic stem cells [1].

**Figure 1:**
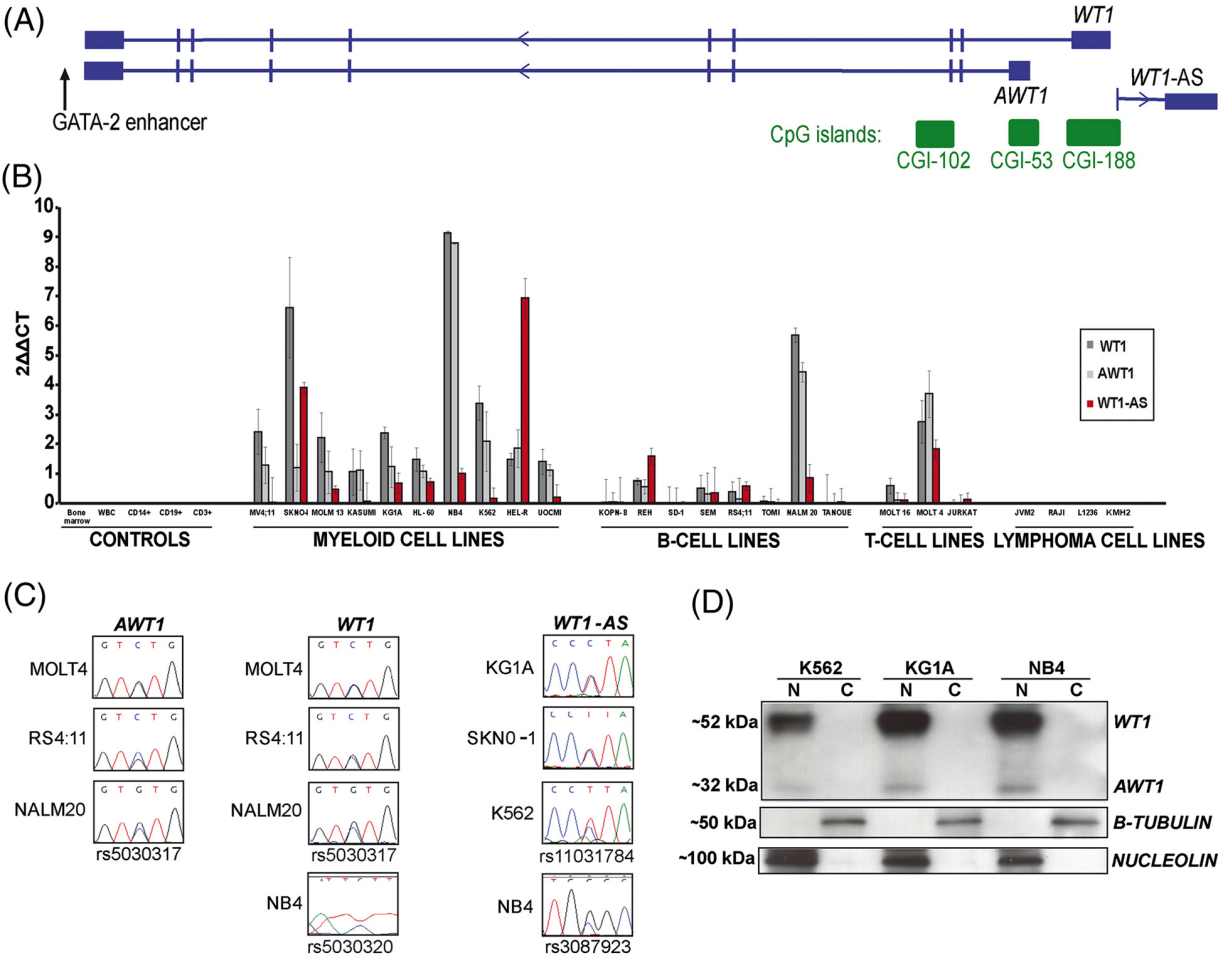
**Expression profiling of the various *WT1 *transcripts in leukemia cell lines. (A)** Map of the *WT1* locus, showing the location of the various promoters, CpG islands and enhancer elements. Arrows represent direction of transcription. **(B)** Expression of *WT1*, *AWT1* and *WT1*-AS in control bone marrow and peripheral blood leukocytes compared to hematological cancer cell lines. **(C)** The allelic expression of the various transcripts in expressing cell lines. **(D)** Western blot analysis of nuclear (N) and cytoplasmic (C) cellular fractionations reveal that the WT1 and AWT1 proteins are nuclear retained in leukemia cell lines. Purity of the cellular fractionations was confirmed using anti-sera against Nucleolin, a nuclear retained protein and cytoplasmic β-tubulin.

Many different isoforms exits for WT1, and it is estimated that it can have over 36 different isoforms generated by alternative transcription initiation, mRNA splicing and alternative translation initiation [2]. The most studied and common splicing events affects exon 5 leading to the presence or absence of 17 amino acids (+17/-17aa) and alternative-splicing within exon 9 with the possible insertion of three amino acids (lysine, threonine and serine: +KTS/-KTS), resulting in a 52-54 kDa protein. Recently an alternative promoter incorporating a unique first exon has been described. The alternative WT1 transcript (*AWT1*) encodes a 32 kDa protein deficient for the first 147 amino acids of the N-terminal of the WT1 protein, which lacks the repressor domain and the RNA recognition motif of the full-length protein [3]. Transcripts from this alternative promoter also undergo splicing events leading to the +/−17aa and +/−KTS isoforms. In addition to these protein-coding transcripts, an non-coding antisense transcript (*WT1*-AS) also originates within the ~5 kb promoter interval (Figure 1A). Both the antisense and *AWT1* transcripts have been reported to be paternally expressed in kidney and blood samples [4,5]. These transcripts originate from a region of partial methylation, the *WT1* antisense regulatory region (*WT1*-ARR) that has been proposed to act as a *cis*-acting transcriptional silencer on the maternal allele [6].

Expression profiling of *WT1* is becoming almost universal in characterizing AML. Recently, raised *WT1* levels at diagnosis and post-induction are associated with poorer outcomes, whereas patients with very low *WT1* level post-intensification had excellent outcomes [7]. Despite aberrant expression of *WT1* being a useful marker for diagnosis and monitoring MRD in AML the underlying epigenetic alterations associated with this expression are unknown [8]. Using a panel of 28 hematological cancer cell lines and more than 350 primary samples we identify AML-specific *AWT1* promoter hypermethylation that is present irrespective of underlying translocation/oncogenic fusion protein or mutations, which is accompanied by a concomitant epigenetic switch at the level of post-translational histone tail modifications. Finally we show that this robust *AWT1* epigenetic signature is an excellent marker for discriminating AML from non-diseased peripheral blood and that this hypermethylation signature can accurately track disease progression, differentiating patients who relapse from those achieving complete remission after allogeneic hematopoietic stem cell transplantation (SCT).

## Results

### Abundant biallelic expression of the *WT1/AWT1* transcripts result in nuclear retained proteins in myeloid derived leukemic cell lines

qRT-PCR was performed on a wide range of leukemia and lymphoma cell lines to evaluate the transcription levels of the *WT1, AWT1* and the non-coding antisense transcript *WT1*-AS. In all myeloid origin cell lines evaluated the *WT1* and *AWT1* transcripts were readily detectable, with high correlation (Pearson correlation, r = 0.90), indicating that they are likely to be co-regulated. The *WT1*-AS transcript was also expressed in most of the myeloid cell lines (Figure 1B). In contrast, the majority of B-, T- cell leukemic cell lines and lymphomas did not express these transcripts, with the exception of MOLT4 and NALM-20, the later being a cell line with a t(9;22) translocation and biphenotypic B-cell and myeloid cell characteristics [9]. The transcripts within the *WT1* locus have been described to be expressed from the paternal allele in an imprinted manner [6,7]. The over-expression of these transcripts in the cell lines allowed us to determine allelic expression. Using primer pairs designed to distinguish individual isoforms (i.e. the forward primer was in the unique first exon of each transcript) and incorporate SNPs identified in the UCSC sequence browser (GRC27/hg19- dbSNP 135) into the final RT-PCR product, we observe that *WT1*, *AWT1* and *WT1*-AS are biallelically expressed in all heterozygous cell lines (Figure 1C). Finally, western blotting using an antibody directed against the C-terminal of the WT1 protein revealed that the abundant RNA levels for *WT1* and *AWT1* isoforms are translated into nuclear retained proteins in the myeloid derived cell lines KG1A, NB4 and K562, consistent with their transcription factor function (Figure 1D).

### *AWT1* promoter hypermethylation despite over-expression in AML

**Figure 2:**
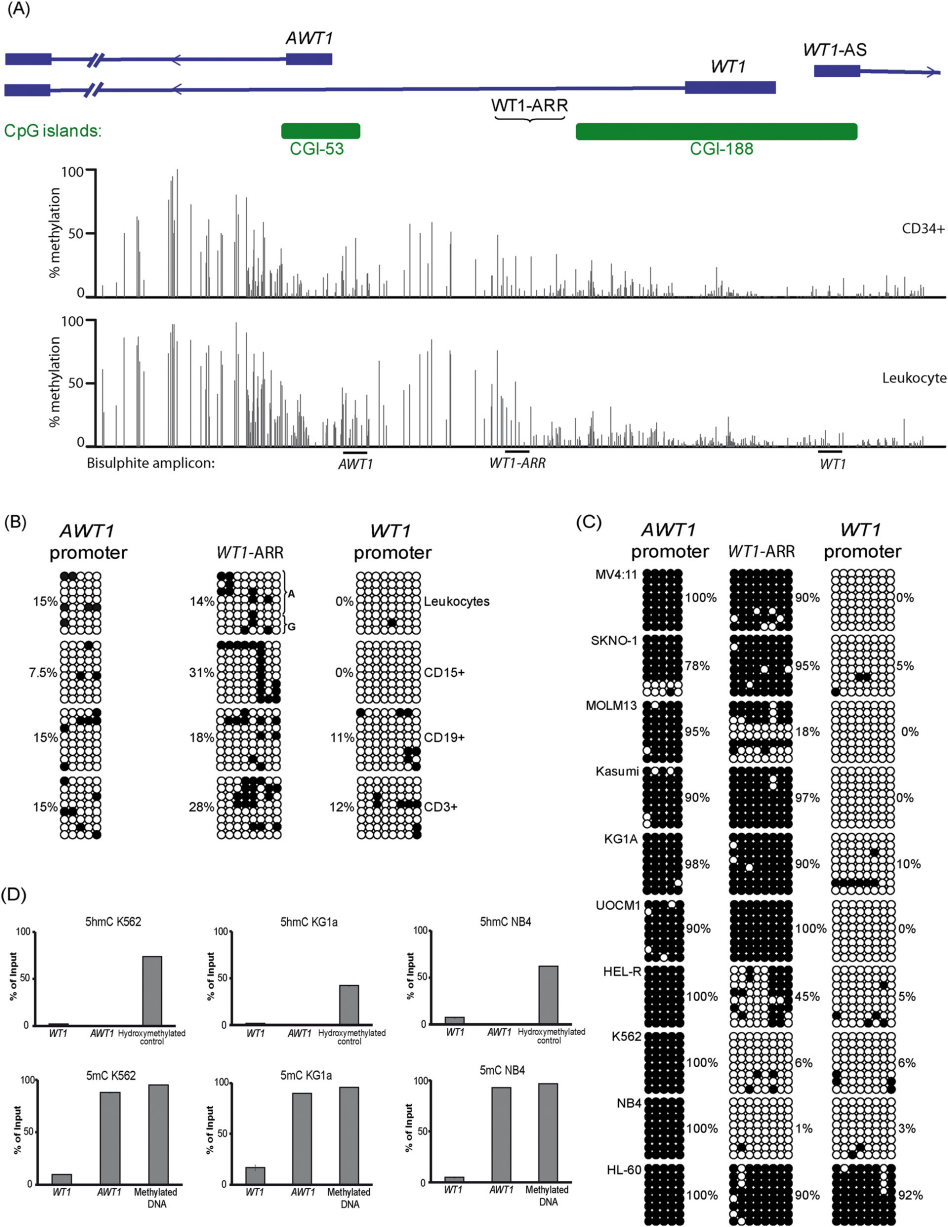
**Methylation analysis in myeloid-derived cancer cell lines. (A)** A detailed map of the *WT1* promoter interval with the methylation profile for CD34+ and leukocyte cells determined by WGBS. Vertical dark grey lines in the WGBS tracks represent the mean methylation value for individual CpG dinucleotides calculated from multiple data sets, with the light grey lines representing the mean + standard deviation. The positions of the bisulphite PCR products are indicated. **(B)** The methylation status of the *WT1* and *AWT1* promoters and the *WT1*-ARR region in control leukocytes and immuno-selected cells. Each circle represents a single CpG dinucleotide on a DNA strand, a methylated cytosine (●) or an unmethylated cytosine (ο). Alrozygous samples. **(C)** The methylation profile in AML cell lines. **(D)** Quantitative PCR on meDIP preciptations using specific antibodies against 5hmC and 5mC in three cell lines that show hypermethylation of the *AWT1* CpG island using bisulphite techniques.

Having confirmed the high expression of *WT1* and *AWT1* in myeloid derived cancer cell lines we next wished to determine if this expression profile was associated with lineage specific DNA methylation changes. We determined the methylation profile of the *WT1 5′* interval using publically available WGBS datasets for CD34+ cells and peripheral leukocytes (Figure 2A). In addition we performed a specific bisulphite PCR analysis across the promoter interval in cell lines, peripheral blood leukocytes and immunosorted CD15+, CD19+ and CD3+ cells.

The promoter region for *WT1* is located within a CpG island of 2.4 kb that is unmethylated in all normal cell types as revealed by the WGBS (chr11: 32454875 32457311 = CD34+ 3% methylated; leukocytes 5% methylated). Approximately 4 kb downstream in intron 1, a second promoter region is embedded within an unmethylated CpG island of 560 bp (chr11: 32452145-32452708 = CD34+ 24% methylated; leukocytes 15% methylated), giving rise to the *AWT1* and *WT1*-AS isoforms (Figure 2A). Adjacent to these promoters is a region of proposed allele specific DNA methylation, the *WT1*-ARR, which has been implicated in regulating expression of these transcripts (chr11: 32454083-32454765 = CD34+ 33% methylated; leukocytes 18% methylated). Our analysis utilizing standard bisulphite PCR and subcloning of normal leukocyte samples heterozygous for the SNP rs11031781 revealed that this region is mosaically methylated on both alleles (Figure 2B), consistent with a lack of imprinted methylation despite normal leukocytes having methylation on the paternal allele at the *H19* loci (Additional file 1: Figure S1A).

Methylation analysis of the entire panel of leukemia and lymphoma cell lines, determined by pyrosequencing, revealed that in myeloid derived cancer cell lines the *WT1* CpG island promoter is unmethylated, except for the HL-60 cell line that was completely hypermethylated, whereas the *AWT1* CpG island promoter was methylated in all cell lines evaluated. The *WT1*-ARR region showed varying amounts of methylation (Figure 2C). In B- and T-cell derived leukemic cell lines the *WT1* promoter was frequently hypermethylated as was the *AWT1* promoter. In lymphoma cell lines, the entire locus was fully methylated (Additional file 1: Figure S1B).

### The *AWT1* methylation detected by bisulphite analysis is 5mC and not 5hmC

Recently 5-hydroxymethylation (5hmC), an oxidized derivative of 5-methylcytosine (5mC) has been suggested to regulate gene expression and is moderately enriched within CpG rich promoters and actively transcribed gene bodies [10]. Unfortunately the deamination of unmethylated cytosines that occurs during conventional bisulfite reactions does not distinguish between 5mC and 5hmC, therefore the bisulphite resistant cytosines we observed in the AML cell lines may reflect enrichment of 5hmC and not 5mC. In order to differentiate these methylation states, we performed DNA immunoprecipitation utilizing antibodies that can discriminate 5hmC from 5mC in the myeloid cell lines, K562, KG1A and NB4. Using this technique we show that the methylation present at the *AWT1* promoter is 5mC (Figure 2D). We confirm these observations using a second method utilizing a highly specific 5hmC glucosyltransferase that tags a glucose moiety to 5hmC but not to 5mC, rendering h5mC refractory to digestion with the Glucosyl-5hmC Sensitive Restriction Endonucleases (GSRE) enzyme (*Msp*I-CCGG) (data not shown).

### *AWT1* hypermethylation is associated with a switch from permissive to repressed chromatin state

**Figure 3:**
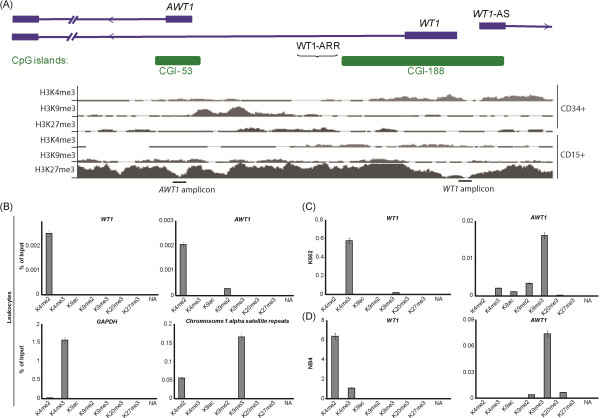
**Histone ChIP in leukocytes and leukemia cell lines.** The histone modification signature determined by ChIP-seq for **(A)** CD34+ and CD15+ cells. The levels of precipitation are comparable as the y-axis is limited to 50 reads for all tracks. The positions of the subsequent PCR amplicons used for ChIP are indicated. **(B)** ChIP specifically targeting the 5′ *WT1* interval in normal leukocytes and myeloid derived cancer cell lines **(C)** K562 and **(D)** NB4. qPCR analysis with primers designed to the *WT1* and *AWT1* promoters performed on immunoprecipitated fractions and mock IPs. Graphs represent the percentage precipitation relative to input chromatin (mean values ± SEM).

Using publically available ChIP-seq datasets from the ENCODE project, we determined the histone modification profile of H3K4me3, H3K27me3, H3K9me3 in normal CD34+ and CD15+ cells. The observed profile was similar to that obtained in control leukocytes using ChIP targeting the *WT1* 5′ interval (Figure 3A, B) suggesting very little cell-type variation in normal blood lineages. Unfortunately no ChIP-seq data is available for H3K4me2 for direct comparison. Subsequent experiments were performed on the K562 and NB4 cell lines that have methylated *AWT1* promoters, unmethylated *WT1* CpG islands with high expression levels of the both transcripts. For these cells lines, the control *GAPDH* promoter and α-satellite repeat regions were normal and comparable to control leukocytes with enrichment of the active H3K4me3 and repressive H3K9me3 marks, respectively. In both cell lines there was massive abundance of the active histone mark H3K4me3 at the *WT1* promoter. This represents a change from modest H3K4me2 enrichment in control leukocytes that may directly reflect the high level of active transcription from this promoter. The *AWT1* promoter had epigenetically switched from an unmethylated interval with H3K4me2 enrichment to having high levels of H3K9me3 in both K562 and NB4, a repressive mark functionally associated with DNA hypermethylation (Figure 3C, D) [11,12]. No H3K27me3 precipitation was observed at the *AWT1* or *WT1* promoter regions in leukocyte or AML-derived cell lines (Figure 3B-D).

### The methylation profiles in cell lines reflect the epigenetic state in primary AML at diagnosis

**Figure 4:**
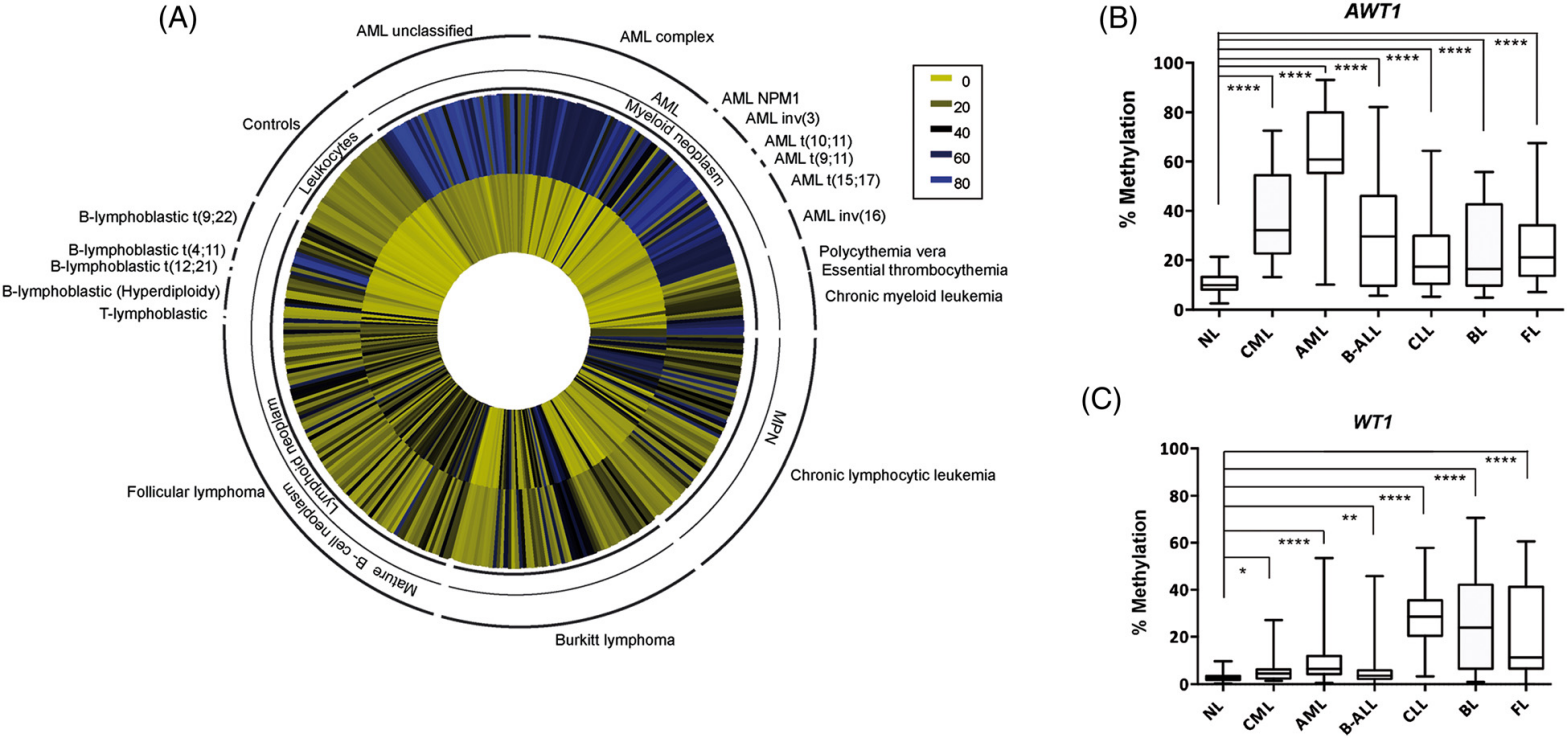
***WT1*/*AWT1 *methylation profiling in primary hematological cancers. (A)** Circular heat map of pyrosequencing results for all samples analyzed indicating disease classification and underlying mutation or translocation. The inner circle represents the methylation values of the *WT1* CpG promoter and the outer circle the *AWT1* promoter. **(B)** Box plot representing the methylation levels at the *AWT1* promoter in normal leukocytes (NL) and in hematological cancers. **(C)** Box plots for the *WT1* methyaltion profiles in the same sample cohort. The differences between groups were evaluated using the Mann–Whitney T-test (*p < 0.05, **p < 0.01, ***p < 0.001, ****p < 0.0001).

To ensure that the methylation profiles obtained in the hematological cancer cell lines could be recapitulated in primary hematological malignancies, we adapted our bisulphite PCRs for quantitative pyrosequencing and assessed a large cohort of cytogenetically well-characterized samples collected at initial diagnosis. These assays gave extremely consistent methylation values in our control cohort with low variability (*WT1* average methylation 2.4%, ± 3SD (0–7.1%); *AWT1* average methylation 15.1%, ± 3SD (5.74-24.4%). However we utilized a strict definition for hypermethylation of >40% to ensure we identify only extreme cases of aberrant methylation. Using this criteria the unmethylated *WT1* CpG island and hypermethylated *AWT1* signature was extremely prevalent in AML patients, including individuals with the recurrent genetic abnormalities inv(16)(p13.1q22), t(16;16)(p13.1;q22) CBFB-MYH11, t(8;21)(q21;p22) AML1-ETO, t(15;17)(q22;q12) PML-RARA, t(9;11)(p22;q23) MLLT3-MLL, t(11; 10), inv(3), inv(9), *NPM1* mutated and unspecified AML (Figure 4A, B). Of the 121 AML patients studied, only 12 samples did not reach this cut off, of which 6 (one inv(3), and 5 complex AML cases) presented with clear hypermethylation compared to controls but >40% methylation which may reflect a lower blast count (Additional file 2: Table S1 and Additional file 3: Table S2). In contrast, only 2 samples had hypermethylation at the *WT1* promoter, both complex cytogenetic AML cases. This analysis revealed that the hypermethylation status of the *AWT1* promoter is an excellent marker, with a positive predictive value (PPV) of 100%, indicating that this marker can correctly detect and classify healthy individual from those with AML. The sensitivity and negative predictive value (NPV) are 86.1% and 89.4% respectively (Additional file 2: Table S3).

### Methylation analysis in other primary hematological malignancies

We extended our methylation analysis to other forms of hematological cancers. Similar to AML, chronic myeloid leukemia cases with BCR-ABL translocations had unmethylated *WT1* promoters and *AWT1* hypermethylation, however, polycythaemia vera and essential thrombocythaemia samples had methylation profiles indistinguishable from normal leukocyte controls. In samples classified with B-cell t(9:22) BCR/ABL, t(4:11) MLL/AFF1, t(12:21) TEL/AML1 and T-cell lymphoblastic leukemias or follicular and Burkitt lymphomas we identified very variable methylation at the promoters, which was often significantly different from normal controls but not reaching the defined 40% methylation cut-off (Figure 4A, C).

### *AWT1* hypermethylation discriminates AML patients who relapse from those achieving complete remission after SCT

**Figure 5:**
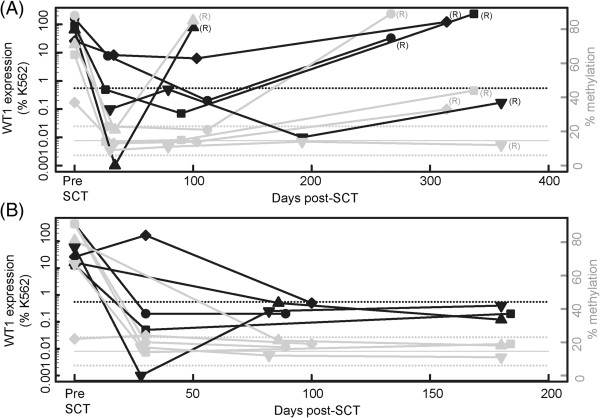
**Time-course of *AWT1 *methylation after SCT.** Evolution of *AWT1* methylation and total *WT1* expression in **(A)** patients who relapsed post-SCT (n = 5) and **(B)** patients in complete remission (n = 4). Black lines represent expression levels relative to the K562 cell line, with the dashed black lines representing the pathological thresholds for expression (0.55% of K562). The grey lines represent the methylation range observed in controls (14.6% ± 3 SD).

Relapse and progression remain the main causes of treatment failure in patients with AML after undergoing allogeneic SCT and often presents as refractory disease. Numerous reports have shown that ~ 90% of patients with *AML* show *WT1* overexpression, therefore, this transcript has been proposed as a useful marker for evaluating MRD after chemotherapy or SCT [13,14]. To determine whether methylation at the *WT1* locus is a useful marker for disease monitoring we performed pyroseqeuncing for the *WT1* and *AWT1* promoters in a series of 9 AML patients before and after allogeneic SCT for whom *WT1* expression had been previously determined [14]. All patients had positive *WT1* expression as determine by RT-PCR at initial diagnosis or in pre-SCT bone marrow samples, of these 5 presented with disease relapse, of which 4 were associated with high *WT1* levels. In 8 cases the *AWT1* promoter region was hypermethylated at initial assessment. During the post-transplantation period, high concordance between normal *WT1* expression levels, *AWT1* hypermethylation and remission status was seen in patients. Specifically, those patients who attained complete remission had *WT1* expression below the cut-off, similar to normal controls, and the *AWT1* region remained unmethylated. In 4 of the 5 patients who underwent disease relapse showed overexpression of *WT1* and gained methylation at the *AWT1* promoter, sometimes to levels higher than at initial diagnosis (Figure 5). Interestingly, the patient with low *WT1* expression at relapse maintained the *AWT1* promoter in an unmethylated state post-SCT, despite being hypermethylated at diagnosis. Interestingly the *WT1* CpG island remained unmethylated (<13% methylation) in all patients and time points (data not shown).

## Discussion

Recent studies have illustrated the power of methylation profiling to identify important cancer-related genes associated with the pathogenesis and classification of hematological cancers. For example *DBC1* hypermethylation is a prognostic marker for AML with normal karyotype [15]. In addition large-scale epigenetic studies have demonstrated that aberrant methylation is a hallmark of specific leukemia sub-types, often associated with known translocations or genetic mutations. This is best highlighted by the AML hypermethylation signature associated with *IDH1/IDH2* mutations, which disrupt TET2 function [16]. In our current study we observe extreme hypermethylation of the *AWT1* promoter, an epigenetic mark generally associated with robust gene silencing, in myeloid-derived hematological cancer cell lines and primary samples. These results are somewhat surprising as high expression levels of the various *WT1* transcripts are frequently observed in AML [17,18], a scenario that is normally mutually exclusive with abundant promoter methylation.

Loss-of-function mutations in *TET2*, an enzyme that converts 5mC to 5hmC have been observed in ~8% of AML cases [19]. Interrogation of the mutation status of *TET2* in the Human Cancer Genome database revealed that Jurkat was the only cell in our panel that had pathological changes (p.Q831H). This suggests that absence of demethylation by mutated TET2 is not associated with the observed *AWT1* hypermethylation.

Previous studies in gliomas and breast tumors have also revealed, contrary to expectation, a high frequency of *WT1* expressing samples with promoter (CpG island 188) hypermethylation [20,21]. Interestingly one study assessed the affect of 5-aza-deoxycytidine on *WT1* expression and observed that despite decreases in methylation there was not a concomitant change in *WT1* expression [21]. Interestingly exposing the KG1A cell line to Decitabine resulted in drastic (>50%) demethylation of the *AWT1* promoter but only a modest doubling in expression levels (Additional file 4: Figure S2). Despite being a specific cancer marker, the tumor-specific methylation at this promoter interval appears to be inconsequential to gene transcription and that other mechanism regulate expression. This implies that when interpreting DNA methylation patterns both cellular context and the genomic position of the CpG sites must be taken into consideration.

Our results in cell lines imply that the *WT1* and *AWT1* isoforms are co-regulated, suggesting they share *cis*-acting regulatory elements. Recently an enhancer region located ~1 kb past the shared 3′UTR has been identified and shown to bind the myeloid-specific transcription factor GATA-2 [22], with abundant expression of *GATA*-2 and *WT1* in AML being associated with bad prognosis [23]. An expression analysis in the 26 of the hematological cancer cell lines and 53 primary AML samples revealed *GATA*-2 and *WT1/AWT1* co-expressed in 96% cases (51/56) consistent with this mechanism being a regulator of expression at the locus (Additional file 5: Figure S3). Computational analysis of the ENCODE dataset reveals that the GATA-2 site is enriched for H3K27ac, a histone modification frequently associated with enhancer elements [24]. This chromatin state may therefore be permissive to chromatin looping between this enhancer and the *WT1* promoter region. Interrogation of the Chromatin Interaction Analysis Paired–End Tags (ChIA-PET) dataset in K562 cells suggests there is a strong physical association between these two genomic elements facilitated by POLII (Additional file 5: Figure S3).

Mutations in *WT1* have been reported in 6-10% of AML cases, 20% of biphenotypic leukemias and sporadic T-cell acute lymphoblastic leukemias (T-ALL) [25-27]. This is a similar frequency to that found in sporadic Wilm’s tumors [28,29]. In AML and T-ALL cases with heterozygous mutations, expression analyses have demonstrated biallelic expression of both the mutated and wild-type *WT1* alleles consistent with our observations that the *WT1* and *AWT1* transcripts are not subject to genomic imprinting [27,30]. Interestingly, direct sequencing of all exons of the *WT1* gene in our panel of cell lines identified only one mutation in addition to known polymorphisms, an A > G nonsense mutation in exon 7 (p.R369*) of the non-expressing U937 cell line.

Despite the plethora of research on the role of *WT1* in leukemia, it remains unclear if the abundant *WT1* levels represent ectopic expression during carcinogenesis or simply reflects the arrested differentiation during early hematopoiesis, since most AML have an immature phenotype. However, the hypermethylation observed at this domain is associated with the tumorgenic changes since methylation at the *WT1* and *AWT1* CpG islands is never observed in any normal hematopoietic cell type or CD34+ stem cells.

The discovery of novel molecular markers that are highly specific and sensitive will improve strategies for both detecting and classifying tumor types, but also in the management of cancer by monitoring a tumors response to novel therapies. Here we show that using a cut-off of >40% methylation at the *AWT1* promoter, the PPV for our assay in AML is 100%, indicating that the analysis can correctly detect and classifies a healthy individual. The sensitivity and NPV are 86.1% and 89.4% respectively, making this an ideal marker to correctly classify AML compared to controls. It must be noted, however, that when assessing all hematological malignancies together, hypermethylation of the *AWT1* promoter it does not perform well as a diagnostic factor for unspecified leukemias/lymphomas (sensitivity 47%; specificity 100%; PPV 100%; NPV 43.6%) (Additional file 2: Table S3). Therefore quantitating *AWT1* hypermethyaltion is ideally suited to monitor MRD in which the diagnosis of AML has already been performed, and the recurrence of the disease during remission needs to be accurately monitored as revealed by our analysis of patients undergoing allogeneic SCT.

## Conclusion

In summary, differences in methylation patterns among tumors may be correlated with clinical features of patients and can serve as markers in cancer classification. Here we describe a methylation signature of the *AWT1* promoter CpG island that is a promising marker for classifying myeloid-derived leukemias. When compared to the results of other well-characterized methylation markers in additional forms of cancer (i.e. *MGMT* in glioblastomas and *GSTP1* in prostate cancers) [31,32] the outcomes are extremely encouraging and further work on larger cohorts, maybe in combination with other leukemia-specific methylation markers, are required to confirm if our observations can be useful in a clinical setting. It remains to be determined what mechanisms are responsible for the correlated up-regulation of *WT1* and *AWT1* expression, with DNA methylation, micro-RNA, enhancer activation or even utilization of an unidentified enhancing long non-coding RNA all possibly having an influence [33].

## Methods

### Patient samples and cell lines

DNA was extracted from frozen peripheral blood and/or bone marrow samples from 356 patients at diagnosis and 102 control peripheral blood samples. For 9 individuals, we also had samples from multiple time points following SCT. Full clinical classification of the cohort can be found in Additional file 2: Table S1 and Additional file 3: Table S2. In addition, DNA and RNA were isolated from 28 derived from various hematological malignancies along with CD15+ (neutrophils and monocytes), CD19+ (B -cells) and CD3+ (T-lymphocyte) cell fractions. The cell lines were purchased directly from the ATCC or DSMZ cell line repositories and were grown in DMEM media with 10% heat inactivated fetal calf serum supplemented with 50 U/ml penicillin and 50 μg/ml streptomycin. In addition all cell lines were routinely tested to ensure they were free of mycoplasm contamination. These studies were approved by the ethical committee of Bellvitge Institute for Biomedical Research/CEIC (PR159/08) and all patients gave informed consent.

### Dataset analysis

To determine the epigenetic landscape of the *WT1* region we analyzed publically available H3K4me3, H3K9me3 and H3K27me3 ChIP-seq datasets and whole genome bisulphite sequencing (WGBS) methylomes for CD34+ and three leukocytes. All data analyses were performed using in-house R-scripts. We subsequently targeted specific regions of interest using bisulphite PCR.

### Methylation analysis

Approximately 50 ng of bisulphite converted DNA was used for each bisulphite PCR or pyrosequencing reaction (for primer sequences see Additional file 2: Table S4). To differentiate between 5′-hydroxymethylation (5hmC) and 5-methylcytosine (5mC) we utilized methylated DNA immunoprecipitations (meDIP) with a specific 5hmC or 5mC antibodies (Diagenode, Liege, Belgium) and the Quest 5hmC Glucosyltransferase Detection KitTM (Zymo).

### Chromatin immunoprecipitations

For ChIP, 100 μg of chromatin was used for each immunoprecipitation reaction with Protein A Agarose/Salmon Sperm DNA and specific antibody raised against various post-translational histone H3 or H4 modifications (H3K4me2 Millipore 07–030; H3K4me3 Millipore 07–473; H3K9ac Cell Signalling 9671; H3K9me2 Millipore 07–441; H3K9me3 Abcam 8898; H4K20me3 Millipore 07–463; H3K27me3 Millipore 07–449). The levels of immunoprecipitated chromatin at specific regions were determined by qPCR using an Applied Biosystems 7900 Fast real-time PCR machine, using SYBR Green and IP levels were compared to Input and the results expressed as percentage of total input material.

### Quantitative RT-PCR

The levels of various *WT1* transcripts were assessed using qRT-PCR. All PCR amplifications were run in triplicate on a 7900 Fast real-time PCR machine (Applied Biosystems) following the manufacturers’ protocol. All primers were optimized using SYBR Green (Additional file 2: Table S4 for primer sequences) and melt curve analysis to ensure that amplicons were specific and free of primer-dimer products. Thermal cycle parameters included Taq polymerase activation at 95°C for 10 min for 1 cycle, repetitive denaturation at 95°C for 15 sec, and annealing at 60°C for 1 min for 40 cycles. All resulting triplicate cycle threshold (Ct) values had to be with 1 Ct of each other. The quantitative values for each triplicate were determined as a ratio with the level of β-Actin expression that was measured in the same sample, and then averaged to provide relative expression values. A cDNA mix of all cell lines and controls was used as an expression calibrator.

### Western blot analysis

We extracted nuclear and cytoplasm protein fractions from cell lines for western blot using the NE-PER Nuclear and Cytoplasmic Extraction kit (Thermo Scientific). SDS-PAGE gel electrophoresis and western blotting was performed as standard. To determine the cellular localization of proteins we incubated resulting membranes with primary antibodies against WT1 (Santa Cruz sc-192), β-Tubulin (Abcam ab6046) or Nucleolin (Santa Cruz sc-8031). Washed membranes were incubated with corresponding peroxidase-conjugated secondary antibody. The immunoreactive proteins were visualized using the ECL Western blotting detection kit (Amersham Biosciences).

## Abbreviations

WT1: Wilms tumour 1 gene; AML: Acute myeloid leukemia; MRD: Minimal residual disease; SCT: Stem cell transplant; RT-PCR: Reverse transcriptase polymerase chain reaction; ChIP: Chromatin immunoprecipitation; 5hmC: 5- hydroxymethylation; 5mC: 5-methylcytosine.

## Competing interests

The authors declare that they have no competing interests.

## Authors’ contributions

AMA carried out the molecular studies and FC performed the bioinformatic analyses. JR, MDO, JS, ZA, AC, ME FP, MJC, IB, MK, RS provided reagents and classified patients. AMA, RS and DM conceived of the study, and participated in its design and coordination and helped to draft the manuscript. All authors read and approved the final manuscript.

## Supplementary Material

Additional file 1: Figure S1 **(A)** Confirmation of imprinted methylation at the *H19*-DMR in peripheral leukocytes. **(B)** Bisulphite PCR analysis of the *WT1* promoter interval in cell lines derived from hematological cancers other than AML. Click here for file

Additional file 2: Table S1 Characterization of hematological neoplasms according to cytogenetic aberrations. **Table S3.** Specificity of *AWT1/WT1* hypermethylation as a biomarker. **Table S4.** The PCR primer sequences used in this study. Click here for file

Additional file 3: Table S2 Characterization of hematological neoplasms using WHO classification with information on age and sex of the patients (if data available). The methylation values obtained following pyrosequencing for the *WT1* and *AWT1* promoters are also given. Click here for file

Additional file 4: Figure S2 **(A)** Methylation values of repetitive DNA elements in the KG1A cell lines treated with different concentrations of Decitabine. **(B)** The methylation values for *WT1* and *AWT1* promoters and **(C)** qRT-PCR for *WT1*, *AWT1* and *WT1*-AS transcripts following Decitabine treatment. Click here for file

Additional file 5: Figure S3 **(A)** Map of the *WT1* locus showing POLII mediated ChIA-PET interactions between the GATA-2 enhancer and the *WT1* promoter interval. **(B)** Abundance of the *GATA*-2 transcription factor in hematological cancer cell lines as determined by qRT-PCR. Click here for file
