# Supplementary figures and images for: Hypermethylation of the alternative AWT1 promoter in hematological malignancies is a highly specific marker for acute myeloid leukemias despite high expression levels
Source: J Hematol Oncol. 2014 Jan 9;7:4. doi: 10.1186/1756-8722-7-4 (PMC3900738; doi:10.1186/1756-8722-7-4)

(A)

Leukocyte

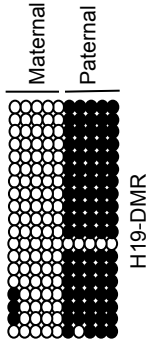

(B)

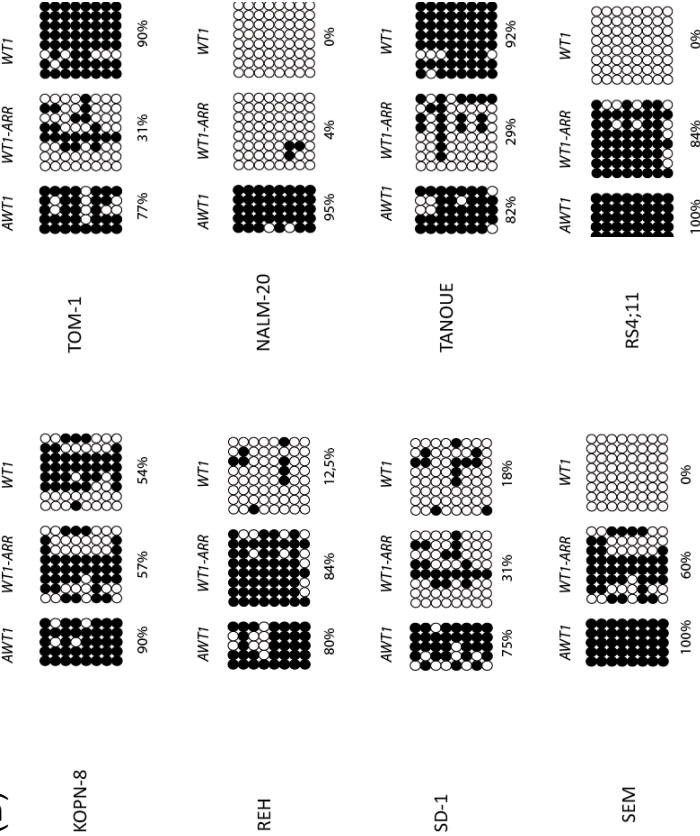

T-cell leukemias

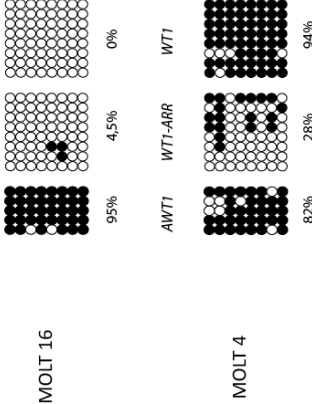

Lymphoma cell lines

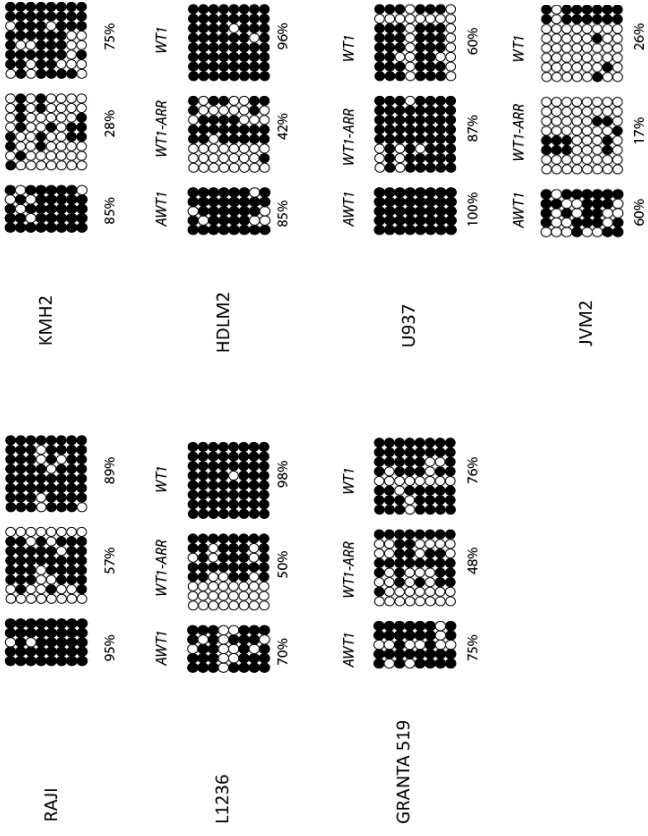

Supplement: Additional file 1: Figure S1 — (A) Confirmation of imprinted methylation at the H19-DMR in peripheral leukocytes. (B) Bisulphite PCR analysis of the WT1 promoter interval in cell lines derived from hematological cancers other than AML. [file 1756-8722-7-4-S1.pdf]

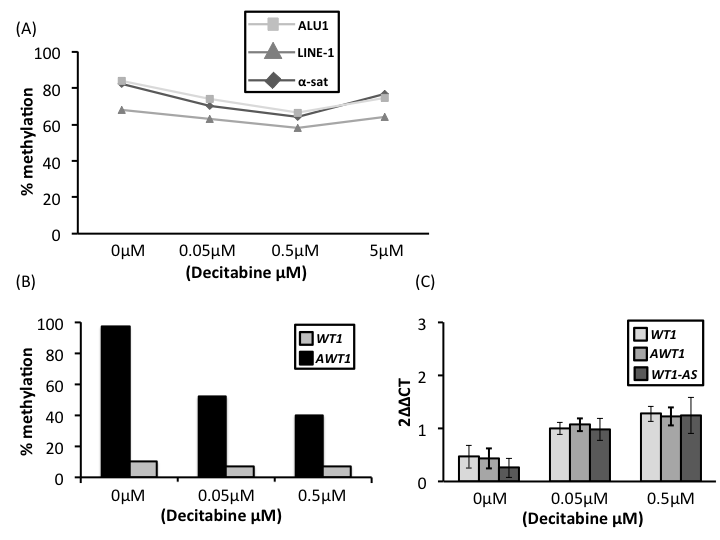

Supplement: Additional file 4: Figure S2 — (A) Methylation values of repetitive DNA elements in the KG1A cell lines treated with different concentrations of Decitabine. (B) The methylation values for WT1 and AWT1 promoters and (C) qRT-PCR for WT1, AWT1 and WT1-AS transcripts following Decitabine treatment. [file 1756-8722-7-4-S4.tiff]

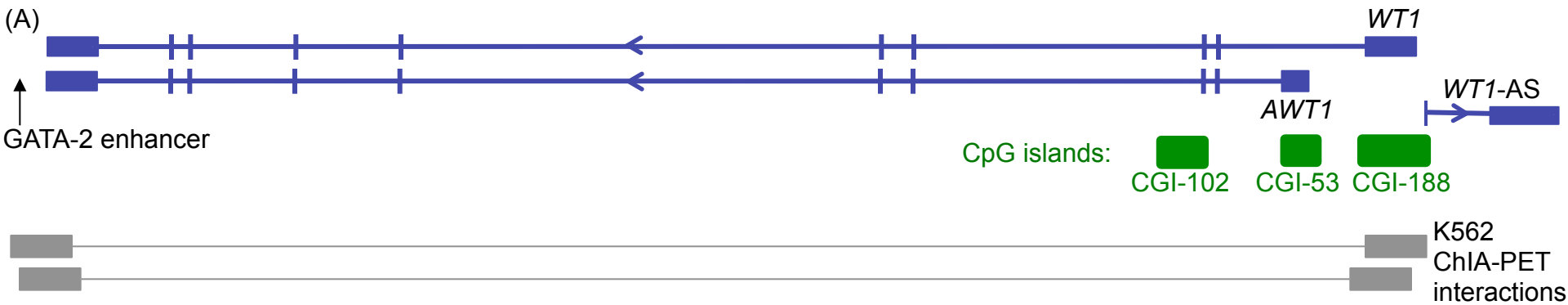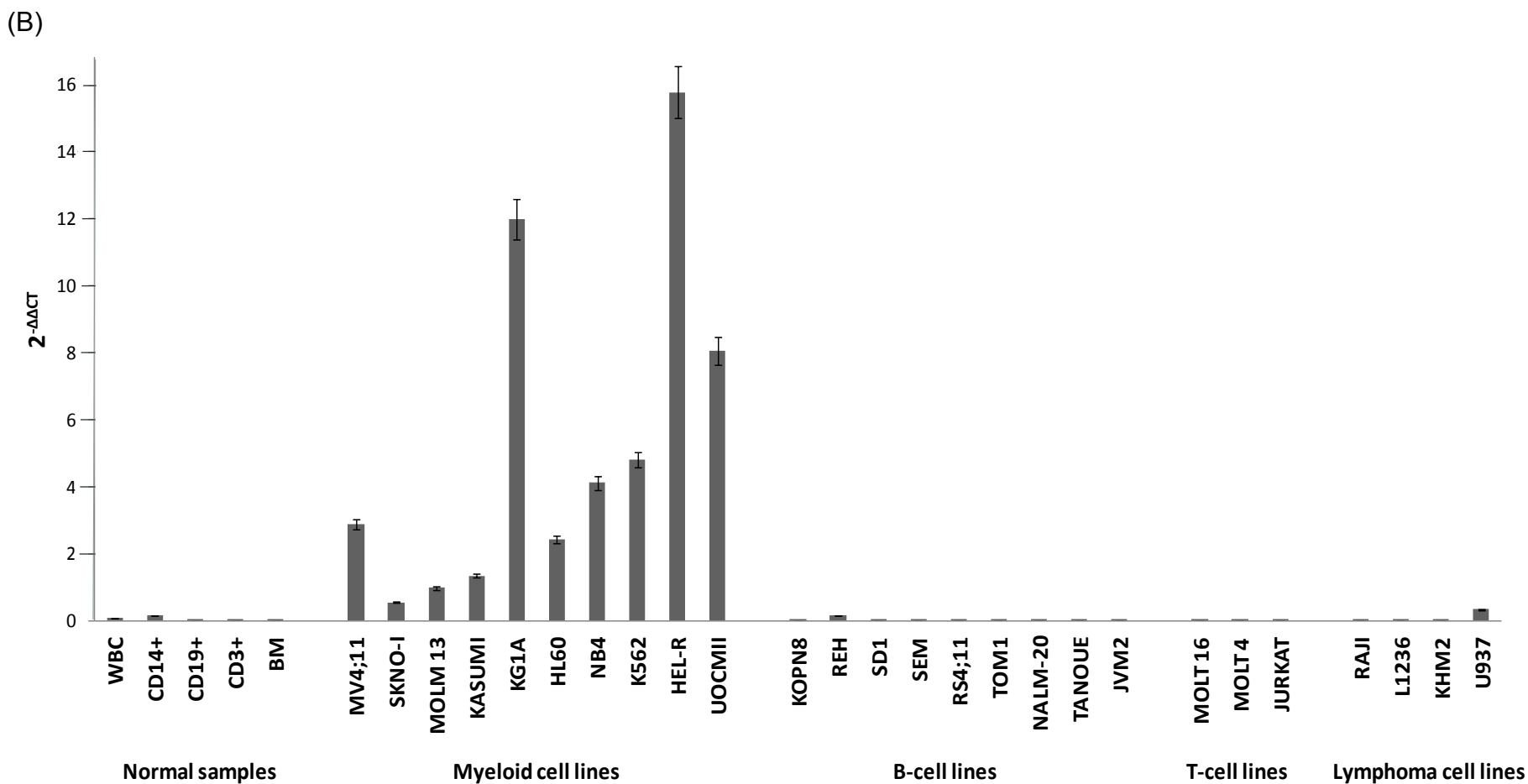

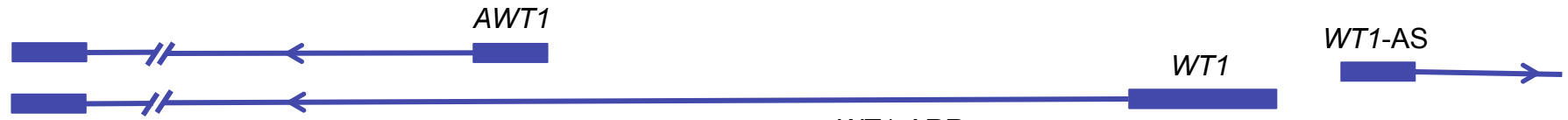

CpG islands:

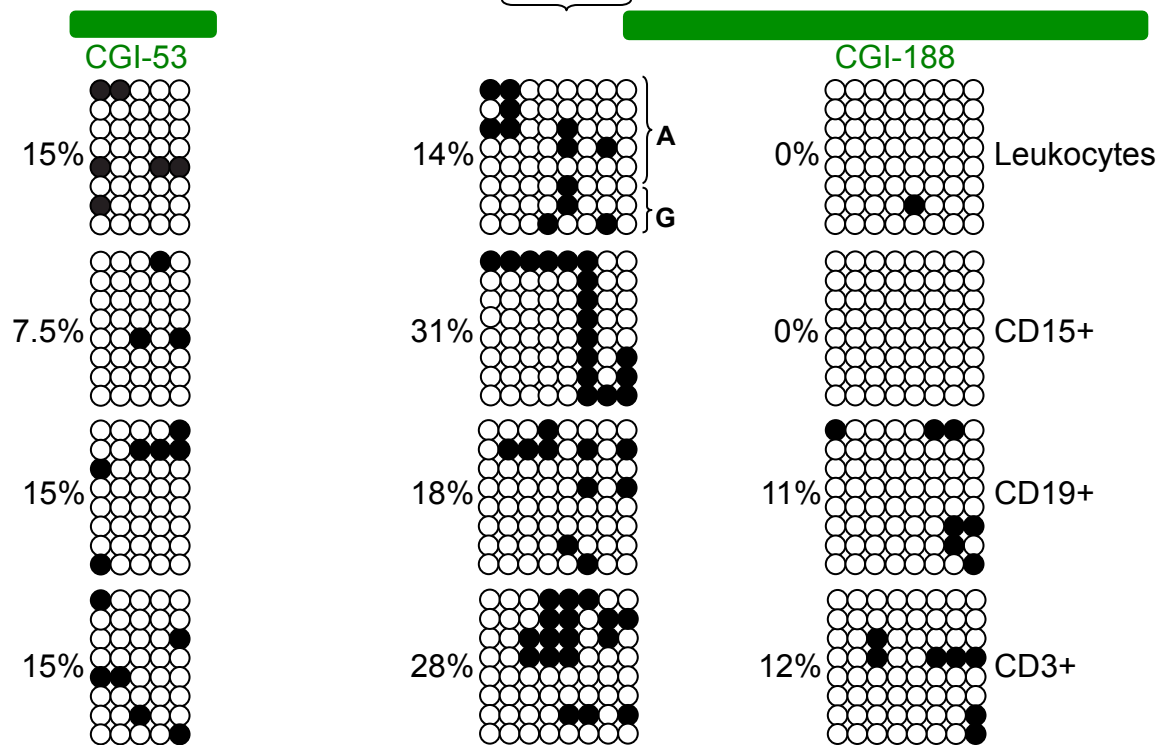

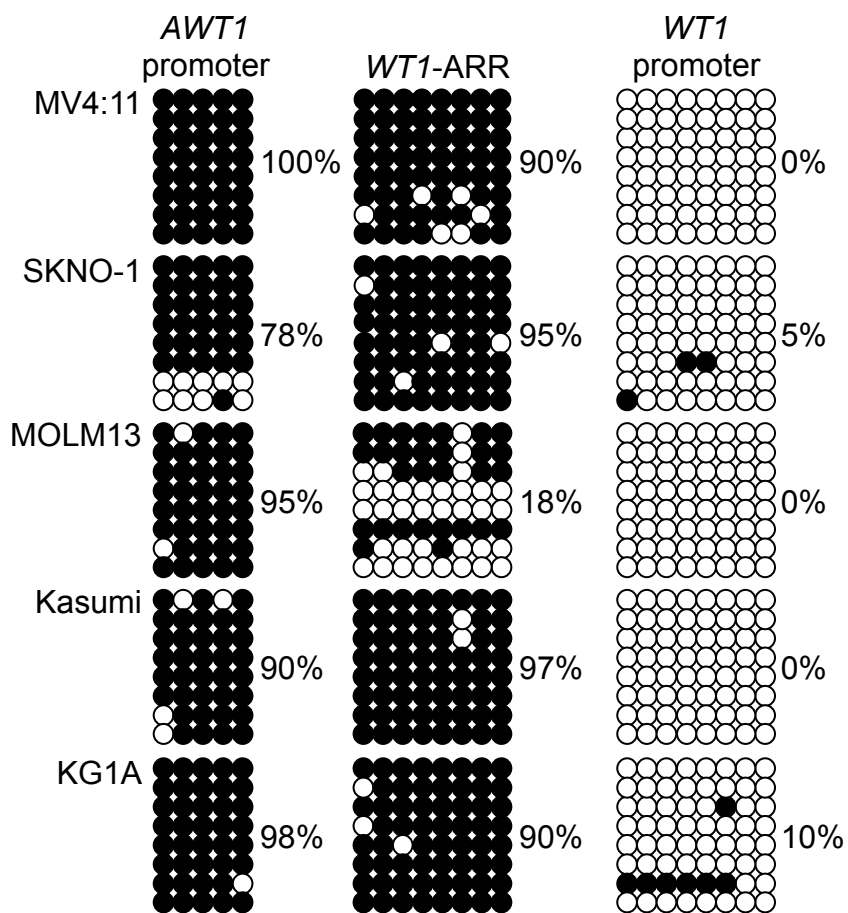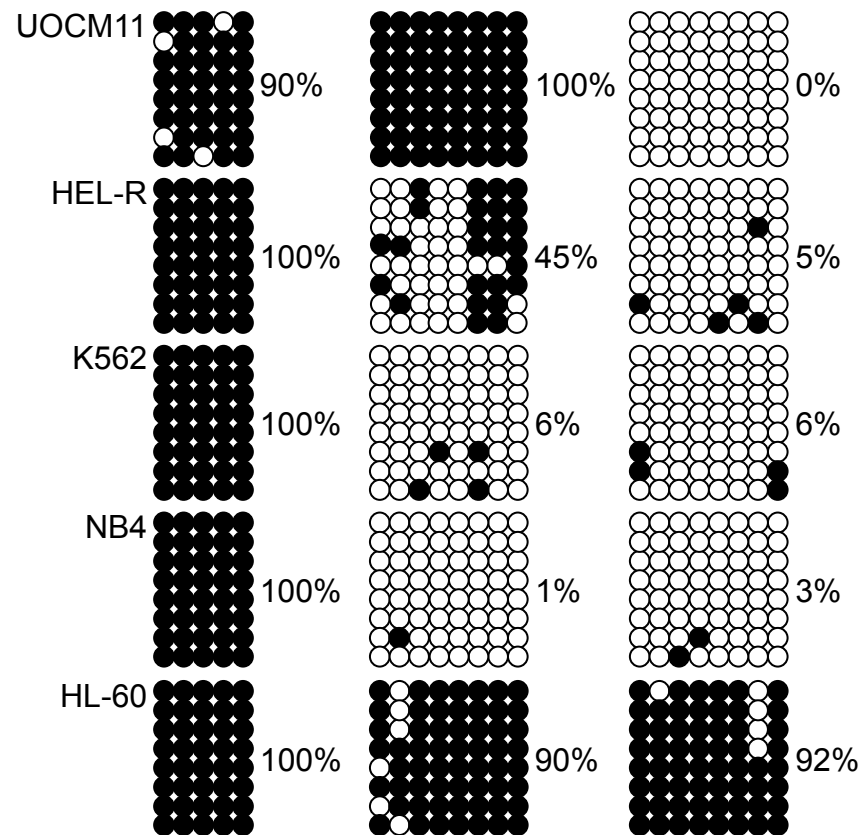

Supplement: Additional file 5: Figure S3 — (A) Map of the WT1 locus showing POLII mediated ChIA-PET interactions between the GATA-2 enhancer and the WT1 promoter interval. (B) Abundance of the GATA-2 transcription factor in hematological cancer cell lines as determined by qRT-PCR. [file 1756-8722-7-4-S5.pdf]
