# Supplementary material for: Hypermethylation of the alternative AWT1 promoter in hematological malignancies is a highly specific marker for acute myeloid leukemias despite high expression levels
Source: J Hematol Oncol. 2014 Jan 9;7:4. doi: 10.1186/1756-8722-7-4 (PMC3900738; doi:10.1186/1756-8722-7-4)
Supplement: Additional file 2: Table S1 — Characterization of hematological neoplasms according to cytogenetic aberrations. Table S3. Specificity of AWT1/WT1 hypermethylation as a biomarker. Table S4. The PCR primer sequences used in this study. [file 1756-8722-7-4-S2.docx]

**Supplemental Table 1:** Characterization of hematological neoplasms.

| **Myeloid neoplasm** | **Number of samples** |
| --- | --- |
| **Myeloproliferative neoplasms (MPS)** |  |
| Chronic myeloid leukemia BCR-ABL1 positive | 12 |
| Polycythaemia vera with JAK2 mutation | 1 |
| Essential thrombocythaemia with JAK2 mutation | 3 |
| **Acute myeloid leukemia (AML)** |  |
| **AML with recurrent genetic abnormalities** |  |
| AML inv(16) | 8 |
| AML t(15;17) | 7 |
| AML t(9;11) | 5 |
| AML t(10;11) | 2 |
| AML inv(3) | 10 |
| AML with *NPM1* mutation | 2 |
| **AML with myelodysplasia-related changes** | 7 |
| **AML not otherwise specified (NOS)** | 70 |
| **Lymphoid neoplasm** | **Number of samples** |
| **Precursor lymphoid neoplasms** |  |
| B-cell lymphoblastic with t(9;22) | 9 |
| B-cell lymphoblastic with *MLL* t(4;11) | 6 |
| B-cell lymphoblastic with t(12;21) | 2 |
| B-cell lymphoblastic with hyperploidy | 9 |
| T-cell lymphoblastic | 1 |
| **Mature B-cell neoplasms** |  |
| Chronic lymphocytic leukemia | 78 |
| Burkitt lymphoma | 39 |
| Follicular lymphoma | 69 |

**Supplemental Table 3:** Specificity of *AWT1/WT1* hypermethylation as a biomarker.

|  | ***AWT1*** | | | | ***WT1*** | | | |
| --- | --- | --- | --- | --- | --- | --- | --- | --- |
| **Disease** | **Sensitivity** | **Specificity** | **PPV** | **NPV** | **Sensitivity** | **Specificity** | **PPV** | **NPV** |
| **Complete cohort** | 47% | 100% | 100% | 43.6% | 26.9% | 100% | 100% | 35.9% |
| **CML** | 33.3% | 100% | 100% | 94.7% | 7.7% | 100% | 100% | 92.3% |
| **AML** | 86.1% | 100% | 100% | 89.4% | 1.64% | 100% | 100% | 54.4% |
| **B-ALL** | 73.1% | 100% | 100% | 95.3% | 3.9% | 100% | 100% | 85.1% |
| **CLL** | 13.9% | 100% | 100% | 67.8% | 86.4% | 100% | 100% | 93.5% |
| **BL** | 27.5% | 100% | 100% | 83.1% | 25% | 100% | 100% | 82.7% |
| **CL** | 20% | 100% | 100% | 71.9% | 25.3% | 100% | 100% | 73% |

**Supplemental Table 4:** Primer sequences

| **PRIMER** | **SEQUENCE** | **PRODUCT SIZE** |
| --- | --- | --- |
| *WT1* Bis F | 5’-Bio-GGTTGAAGAGGAGGGT | 197bp |
| *WT1* Bis R | 5’-GAGTTTTGGGAAGTTGAGGG |  |
| *WT1* Bis Pyrosequencing | 5’-GGTTTGGGTTGTTGAGTGAATGG |  |
| *AWT1* Bis F | 5’-Bio-TAGGAAGGTGTTTAGAAGGTT | 187bp |
| *AWT1* Bis R | 5’-GGTTTAATTATGTTTTAGGG |  |
| *AWT1* Pyrosequencing | 5’-TATTTGATTTTAGGGTGG |  |
| *WT1* qRT-PCR F | 5’-CGCTATTCGCAATCAGGGTTAC | 125bp |
| *WT1* qRT-PCR R | 5’-ATGGGATCCTCATGCTTGAATG |  |
| *AWT1* qRT-PCR F | 5’-GAGAAGGTTACAGCACGGTCAC | 109bp |
| *AWT1* qRT-PCR R | 5’-ATGGGATCCTCATGCTTGAATG |  |
| *WT1-AS* qRT-PCR F | 5’-AGAGTCCGTTCAGAATCCTTG | 123bp |
| *WT1-AS* qRT-PCR R | 5’-AGGCTGCAGGGAACTCCTCCCA |  |
| *GATA-2* qRT-PCR F | 5’-GGCTCGTTCCTGTTCAGAAGGC | 164bp |
| *GATA-2* qRT-PCR R | 5’-TGGTCGGTTCTGCCCATCATCTGT |  |
| *β-ACTIN* qRT-PCR F | 5’-CCTGACGGCCAGGTCATCAC | 155bp |
| *β-ACTIN* qRT-PCR R | 5’-GGAGCAATGATCTTGATCTTC |  |
| *GAPDH* qRT-PCR F | 5’-CGTATTGGGCGCCTGGTCACC | 180bp |
| *GAPDH* qRT-PCR R | 5’-CACCACCTTCTTGATGTCATC |  |
| *WT1* CpG GSRE/ChIP F | 5’-CCTCCGGCCCTGGAGACGTTCAGC | 174bp |
| *WT1* CpG GSRE/ChIP R | 5’-TTGCTGCAGGACCCGGCTTCC |  |
| *AWT1* CpG GSRE/ChIP F | 5’-CCTTCCTGGCTGAGCCTGCTGCTGTC | 188bp |
| *AWT1* CpG GSRE/ChIP R | 5’-GGTAGCGCCTTTCCCACGGTTAGTC |  |
| GAPDH ChIP F | 5’-TAGTGCGCAGCGGGTGCATC | 181bp |
| GAPDH ChIP R | 5’-GCTAGCTCGCTCCACCTGACTT |  |
| HS-SAT ABCAM F | SEQ NOT PROVIDED |  |
| HS-SAT ABCAM R | SEQ NOT PROVIDED |  |
